# Supplementary material for: The implementation and effectiveness of multi-tasked, paid community health workers on maternal and child health: A cluster-randomized pragmatic trial and qualitative process evaluation in Tanzania
Source: PLOS Glob Public Health. 2023 Sep 19;3(9):e0002050. doi: 10.1371/journal.pgph.0002050 (PMC10508634; doi:10.1371/journal.pgph.0002050)
Supplement: S1 Table — (DOCX) [file pgph.0002050.s002.docx]

**S1 Table**

**Socio-demographic balance of baseline sample (2011)**

| **Outcome** | | **Baseline (2011)** | | |
| --- | --- | --- | --- | --- |
| *Mother-level indicators* | | *Intervention (n=454)* | *Comparison (n=428)* | *p* |
| 1 | Age (mean, range) | 27 (15, 46) | 28 (15, 48) | <0.001 |
| 2 | Educational attainment (%, n)  Did not study  Primary only  Some secondary and higher | 27 (123)  70 (318)  3 (13) | 30 (129)  66 (283)  4 (16) | 0.34 |
| 3 | Marital status (%, n)  Married  Not married | 85 (386)  15 (68) | 83 (355)  17 (73) | 0.11 |
| 4 | Wealth status (quintile) (%, n)  First  Second  Third  Fourth  Fifth | 21 (95)  22 (100)  28 (127)  18 (82)  11 (50) | 16 (68)  23 (98)  27 (116)  19 (81)  15 (65) | 0.07 |
| 5 | Parity (mean, range) | 3.6 (1, 12) | 3.8 (1, 14) | <0.001 |
| 6 | Distance to nearest health facility  <5 kilometers  5-10 kilometers  ≥10 kilometers | 49 (222)  38 (173)  13 (59) | 52 (223)  36 (154)  12 (51) | 0.37 |
| *Child-level indicators* | | *Intervention (n=1038)* | *Comparison (n=1066)* |  |
| 1 | Age (months) (mean, range) | 32 (4, 59) | 34 (3, 57) | 0.54 |
| 2 | Sex  Male  Female | 50 (514)  50 (524) | 50 (537)  50 (529) | 0.41 |
| 3 | Primary caregiver  Parent  Other | 94 (972)  6 (66) | 94 (1004)  6 (64) | 0.79 |
