# Supplementary material for: The implementation and effectiveness of multi-tasked, paid community health workers on maternal and child health: A cluster-randomized pragmatic trial and qualitative process evaluation in Tanzania
Source: PLOS Glob Public Health. 2023 Sep 19;3(9):e0002050. doi: 10.1371/journal.pgph.0002050 (PMC10508634; doi:10.1371/journal.pgph.0002050)
Supplement: Attachment — Submitted filename: Response to Reviewers.docx [file pgph.0002050.s003.docx]

Response to reviewers of : **“The implementation and effectiveness of multi-tasked, paid community health workers on maternal and child health: A cluster-randomized pragmatic trial and qualitative process evaluation in Tanzania”**

**Table 1: Editor Feedback and Authors’ Response**

| **Editor review comments** | | |
| --- | --- | --- |
| 1 | Please include an explanation for the retrospective CT registration and confirmation that all related CTs are registered. | To complete CT registration, we were asked to provide an individual participant data sharing plan. To obtain permission for sharing individual participant data, at the time, it was required to obtain a 'materials transfer agreement' (MTA) from the National Medical Research Coordinating Committee of the National Institutes of Medical Research of Tanzania (NIMR). We also needed to obtain the MTA to follow a donor requirement that all study data be submitted to a central repository where the donor would eventually make the study data publicly available. We submitted our MTA application to NIMR immediately after the NIMR ethics review committee approved the study in early 2011. The application remained pending for approximately one-year and was eventually not approved. Once it became clear that we'd not obtain the MTA, we went forward with applying for CT registration. Our registration application succeeded despite the absence of an individual participant data plan but unfortunately this occurred after the trial started. However, the study received formal approval from the Tanzanian and US-based ethical review communities in 2011, well before the launch of any trial procedures. We confirm that all related CTs are registered. |
| 2 | In the online submission form you indicate that your data is not available for proprietary reasons and have provided a contact point for accessing this data. Please note that your current contact point is a co-author on this manuscript. According to our Data Policy, the contact point must not be an author on the manuscript and must be a third party. Please revise your data statement to a non-author institutional point of contact, such as a data access or ethics committee, and send this to us via return email. Please also include contact information for the third party organization, and please include the full citation of where the data can be found. | The coauthors discussed the issue of Data Availability with staff of the Ifakara Health Institute’s Data Repository and Ethics Review Committee. These individuals are points of contact with the Ethics Review Committee of the National Institute of Medical Research of Tanzania. Both Ethics Review Committees (IHI and NIMR) approved the trial protocol. During our discussions we became aware of a 2023 publication in *PLoS Global Public Health* in which authors from the Ifakara Health Institute suggested that interested parties either request data access from the corresponding author or a point of contact that was a non-author from the Ifakara Health Institute. This article is:  Mtenga S, Mhaliu G, Ostensky B, Ramaiya K, Kassim T, Hooley B, et al. (2023) Social-political and vaccine related determinants of COVID-19 vaccine hesitancy in Tanzania: A qualitative inquiry. PLoS Glob Public Health 3(6): e0002010. <https://doi.org/10.1371/journal.pgph.0002010>.  It is our preference that use a similar statement for our paper since it was approved by *PLoS Global Health* for a publication from the same research institute earlier this year. The revised data available statement that we propose is:  The ethical approvals from the Ethical Committee at the National Institute for Medical Research (NIMR) in Tanzania and the Ifakara Health Insitute do not give us license to make the data set publicly available. However, data may be made available on request to the Ifakara Health Institute Ethics Committee contact person: Dr Mwifadhi Mrisho (mmrisho@ihi.or.tz) at the department of Health System Impact Evaluation, Plot 463, Kiko Avenue, Mikocheni, PO Box 78373.  When referencing the data please use the following citation: Phillips JF, Hingora A. Introducing Community Health Agents to Accelerate Achievement of MDG 4 and 5 in Tanzania - The Connect Project. The Ifakara Health Institute, Dar es Salaam, Tanzania. Available from: <https://data.ihi.or.tz/index.php/catalog/Impact-evaluation/about.> |
| 3 | Please provide a/amend your detailed Financial Disclosure statement. This is published with the article. It must therefore be completed in full sentences and contain the exact wording you wish to be published.  State the initials, alongside each funding source, of each author to receive each grant.  If you did not receive any funding for this study, please simply state: “The authors received no specific funding for this work.” | We have reviewed the financial disclosure statement and feel that we used complete sentences. We reviewed other financial disclosure statements from other recent *PLoS Global Public Health* publications, and suggest that the following be used for our publication:  This study was funded through a joint funding initiative by the United States based Doris Duke Charitable Foundation through grant number DDCF2009058a (JFP) and the United Kingdom based charity Comic Relief through grant number 112259 (KT). The funders had no role in designing or implementing the program that was evaluated through this study, nor in the design of the study, data collection and analysis, decision to publish or preparation of the manuscript. |
| 4 | We have noticed that you have uploaded Supporting Information files, but you have not included a list of legends. Please add a full list of legends for your Supporting Information files after the references list. | We moved the list of supporting information files from after the conclusions to after the references. Please see the tracked changes on page 47 of the revised manuscript. |
| 5 | Potential copyright issues regarding Figure 1, the map of the study areas. | Coauthors discussed journal requirements for publishing maps and the relative value that the Figure 1 map adds to our manuscript, which already has several other figures. We concluded that we should remove Figure 1 from the manuscript. Please see tracked changes on page 6 of the revised manuscript. |

**Table 2: External Reviewers Comments and Authors’ Response**

| **Reviewer 1** | | |
| --- | --- | --- |
| 1.1 | P6: Figure 1 , P11: Figure 2 P 23, p 26, p 29, all those figures are announced in the text but not shown. A decision need to be made to insert the figures in the text or to leave them as an annex | The Editor instructed us to ignore this comment. |
| 1.2 | P 14 Method: This sentence may be confusing : “We purposively sampled four nodes in Ifakara and two in Rufiji. We sampled two urban nodes in Ifakara and Rufiji, respectively, and four rural nodes.” Is it 1 urban node in Ifakara and 1 in Rufiji? Make this part clearer. Additionally, when presenting the study setting, it will help to announce that some of the sites are urban , others rural and identify in what districts they are. | Thank you for the comment. We have included a sentence on page 6, lines 133-135 in the description of the study setting to indicate that the populations were predominantly rural, however that there were small urban areas. On page 14 (lines 319-320), we amended the wording of the sentences the reviewer pointed out to provide better clarity on the qualitative sample. |
| 1.3 | P16: It could have been interesting to know how the result of the quan and qual linkage will be presented. | We added a brief paragraph (two sentences) on lines 370-374 of page 17 to clarify how the quantitative and qualitative results will be presented. |
| 1.4 | P17: “There is some indication that the intervention might have inhibited utilization of ANC and PNC at clinics, possibly since WAJA performed the information, education, and counseling components of these services in households, though all findings are statistically insignificant.” This segment highlighted in red will fit better in the discussion part than the results section. | We have made the recommended revision. The sentence the reviewer pointed out on page 17 has been removed and re-introduced to the revised version of this manuscript in the paragraph that starts the discussion section, lines 238-241 on page 30. |
| 1.5 | P 31 in the sentence “and was, thus, was a notable barrier to acceptability”. Delete one of the “was” | Thanks for catching this error. We have made the needed revision (line 263, page 31). |
| 1.6 | Considering the study limits, does the fact that the study participants was not blinded about their treatments can influence the evaluation results? | Because participants were not blinded to their treatment status, and comparison communities were near to intervention communities, it is possible that members of comparison communities came to intervention communities to receive care from WAJA. This might have biased our quantitative results. We introduce this as a study limitation in the revised version of the manuscript on lines 335-338 on page 35. |
| **Reviewer 2** | | |
| 2.1 | Authors report that the ‘community health worker program reduced incidence of childhood illness and improved access to timely and appropriate sick childcare; however, there was no effect on maternal, newborn and child health service utilization’—this statement needs be clearly explained as this can add confusion to readers. How is childhood illness incidence reduced when there was no effect on service utilization? | We believe that this sentence was in the abstract. We have revised the language to promote better clarity. Please see the sections of the abstract which have tracked changes in our revised manuscript. Indeed, the improved outcomes were among all children under-five, whereas the lack of impact was observed with respect to facility-based utilization of maternal and newborn health care services (not services for all under-five children). Therefore, we report that the community-based interventions conducted by WAJA in the community (e.g., counseling to parents and caretakers and treatment for basic childhood illnesses) led to positive outcomes for under-five children; however, weaknesses and lapses in availability of quality ANC, delivery care, and care for newborns in the wider health system led to failure in the referral component of the WAJA intervention. Because of this, while WAJA implementation was able to improve child health in communities, it did not increase access and utilization of services for mothers and newborns at facilities. |
| 2.2 | And then at line 55: Authors statement reads ‘The lack of……among community health workers’. You have attributed it to lapses in the availability of needed care at facilities and or working materials among community health workers. So, it could be a major limitation compromising the outcomes including reduced childhood illnesses? In other words, Why did this factor not affect the childhood illnesses? | This abstract has been revised to impart an accurate summary of our findings. Indeed, while the qualitative research did reveal that WAJA did periodically experience stock outs, we found that despite this they managed to sustain their motivation and commitment to the program. In the end, our analysis of survey data found that they were able to reduce incidence of childhood disease and access to sick child care despite occasional lapses in their implementation readiness during the last half of the trial (see pages 26-27). The lack of program effects that the reviewer has pointed out (abstract, line 55) concerned utilization of facility-based maternal and newborn services, not under-five outcomes (see the tracked changes in the abstract where we clarify this distinction). Failure to improve maternal and newborn service utilization at facilities was due to the weak health system in which important, high quality maternal-newborn health care services were frequently unavailable, not due to WAJA stock outs.  Yet, the reviewer asks an interesting question about how WAJA deployment could have improved child health outcomes even though there were periodic stock outs in the last half of the trial. Four points might help explain this: First, as we state in our methods section, the baseline and endline household surveys measured the incidence of childhood morbidity and access to sick child care by interviewing caregivers about the wellness of their under-five children and care-seeking behaviors during the two-weeks prior to each survey. It is possible that, during the endline survey, this recall period did not coincide with times when there were WAJA stock outs. If this is so, then our study would not have captured the effects of WAJA-level stock outs, which occurred periodically between 2013-15. Note that in our 2019 publication of the WAJA impact on child mortality, our main outcome, we measured program impact using longitudinal demographic surveillance data (<https://link.springer.com/article/10.1186/s12913-019-4203-1>).  In this paper, our analysis did capture the effects of WAJA stock outs over time during the years of the trial, which we argue was the driver behind the failure of the program to improve child survival. Second, to prevent childhood illness WAJA only required job aids which they used to guide their provision of counseling and education to caregivers on ways to prevent and respond to childhood illness if/when it occurs. They did not require any medications, which were only needed if WAJA encountered children who were already sick. Because stock outs were limited to medicines and supplies, but not job aids, WAJA could still have effectively prevented childhood illness in the community even when there were stock outs. Third, the protocol was for WAJA to refer children to facilities if children present to WAJA with a symptom or condition that WAJA could not treat, and frequently to ensure referral compliance, WAJA would accompany children to the facility. Therefore, even when stocked out, WAJA were still able to identify sick children in a timely manner and assist them in receiving needed care from facilities if they did not have medications themselves. Fourth, if ever WAJA were unable to treat a sick child and felt that the nearby facility may also be stocked out of essential supplies, WAJA would use mobile phones supplied to them by the project to call their fellow WAJA that might have needed medications (WAJA were able to communicate with each other for free within a 'closed user group' that was paid for by the project). Once they found a fellow WAJA that had needed medications, WAJA who were stocked out could receive medications from another that was not and use this medicine to ensure that sick children received appropriate care as soon as possible. These explanations may help the reviewer understand why our paper did not identify any impacts of WAJA stock outs on childhood illness. |
| 2.3 | Since the CHW deployment also has heterogeneity in terms of who they are and how they could affect the outcome, did you account the individual differences, their aptitude, performance, and catchment area impacts? | No, we did not do so in this analysis. We have reported on variation on WAJA aptitudes and performance and quality of care in a cross-sectional study we performed to determine whether WAJA could perform integrated community case management for childhood illness as well as 'gold standard providers' (<https://www.ncbi.nlm.nih.gov/pmc/articles/PMC6358652/>).  As we were only able to capture measures of WAJA aptitude and performance cross-sectionally, we are not comfortable generalizing these individual attributes of WAJA seen at moments in time to a community-level measure that reflects the entire duration of the trial. Doing so, and bringing such a measure into our analysis, we feel, would have introduced significant measurement bias and, it is likely, resulted in spurious results. |
| 2.4 | It looks like the CHW training and interventions experienced a lot of changes in terms of supervision, did that affect the outcome? Has that been accounted, particularly on how that may have had impact on their performance and ultimately outcomes? | The section that presents the qualitative results includes a discussion on how contextual factors caused some variation in the quality and consistency of supportive supervision of WAJA and relates this discussion to proximal and distal outcomes (see pages 26-28 and Figure 6). In addition, some of authors of this paper are coauthors on other papers written using data from the Connect process evaluation, which elaborate on the same issue in relation to the quality of sick child care performed by WAJA (https://www.ncbi.nlm.nih.gov/pmc/articles/PMC6358652/), WAJA time use and productivity (<https://bmchealthservres.biomedcentral.com/articles/10.1186/s12913-016-1718-6>) and stakeholders' perception of the feasibility and effectiveness of the WAJA program (<https://www.tandfonline.com/doi/full/10.1080/17441692.2015.1080750?scroll=top&needAccess=true&role=tab>). |
| 2.5 | Also can you explain why IHI withdrew from supporting implementation? | As explained in the introduction, the evidence base on CHW programs is in need of findings from randomized studies of CHW interventions that are delivered through routine health systems (page 5 line 115-117). In addition, we report that the Government of Tanzania commissioned our evaluation of the WAJA program to generate systematic knowledge on whether a CHW program would be acceptable, feasible and have an incremental impact of maternal and child health if implemented by the Tanzanian health system (page 7, lines 154-157). We then state that "implementation arrangements of Connect balanced the overriding goal of understanding the effects of WAJA deployment through the routine health system with the reality that to deliver the program, the health systems required support from development partners" (page 9 lines 194-196) and describe the numerous steps the IHI took to work with district health officials, adapt the intervention to local health system requirements and conditions, and launch the program. This occurred in the first two years of the trial during which time we sought to build districts' capacity to adopt and implement the program independently. We explain that this type of collaboration ended after the second year of trial when the IHI withdrew implementation support, so that the evaluation would be able to report on the effects of the program after it transitioned to the districts. This is consistent with our mandate to conduct a pragmatic trial and fill gaps in the global and Tanzanian evidence base on CHW programs. |
